# A Pilot Study on Attentional Focus in Prescribing Physical Exercise in Outpatients with Obesity

**DOI:** 10.3390/healthcare10112306

**Published:** 2022-11-17

**Authors:** Luca Cavaggioni, Luisa Gilardini, Gabriella Redaelli, Marina Croci, Raffaella Cancello, Paolo Capodaglio, Amalia Bruno, Simona Bertoli

**Affiliations:** 1 Department of Biomedical Sciences for Health, Università degli Studi di Milano, 20129 Milan, Italy; 2 Obesity Unit and Laboratory of Nutrition and Obesity Research, Department of Endocrine and Metabolic Diseases, IRCCS Istituto Auxologico Italiano, 20145 Milan, Italy; 3 Orthopaedic Rehabilitation Unit and Research Lab for Biomechanics, Rehabilitation and Ergonomics, Istituto Auxologico Italiano IRCCS, San Giuseppe Hospital, 28824 Piancavallo di Oggebbio, Italy; 4 Department of Surgical Sciences, Physical Medicine and Rehabilitation, University of Turin, 10126 Turin, Italy; 5 International Center for the Assessment of Nutritional Status (ICANS), Department of Food, Environmental and Nutritional Sciences (DeFENS), University of Milan, 20133 Milan, Italy

**Keywords:** obesity, attentional focus, external focus, physical exercise

## Abstract

This pilot study compared the effects of two attentional focus strategies on fitness parameters and body composition in outpatients with obesity. This was a randomized, controlled study that enrolled 94 obese individuals and allocated them into an internal focus group (IF) or an external focus group (EF) while performing six weeks of a home-based training program. The home-based exercise program was the same for both groups except for the instructions that shifted the attention to an external or an internal condition. At the beginning and after the intervention period, participants were assessed for functional performance using the Functional Movement Screen (FMS), body balance using the Modified Balance Error Scoring System (M-BESS) and muscular strength with the Handgrip Strength Test (HST) and the Five-Repetition Sit-To-Stand (FRSTS) test. Concerning body composition and anthropometric parameters, the body mass index (BMI) and fat mass percentage (FM%) were calculated. Significant improvements, main interactions and effects of time and groups were highlighted in the EF group as compared to the IF group in FMS (35% vs. 21%), M-BESS (42% vs. 18%), HST (13% vs. 7%) and FRSTS (23% vs. 12%) measures, while FM% (5%) and BMI (6% vs. 5%) showed a similar improvement overtime (*p* < 0.001). In conclusion, our findings provide initial evidence that a 6-week training program performed following external focus instruction is able to promote significant enhancements in movement efficiency, balance and muscular strength as compared to an internal focus cue. Fitness coaches and therapists might consider integrating a specific attentional focus strategy when designing rehabilitation programs in subjects with obesity.

## 1. Introduction

The regular practice of physical exercise has been considered beneficial for health and wellbeing thanks to different bodily adaptations in cardiorespiratory, neuromuscular and metabolic functions [1]. When it comes to planning an effective training program, the International Guidelines highlight the correct manipulation of different parameters (i.e., volume, intensity, load, exercise order, exercise selection, velocity of muscle action and rest periods) for wellness or performance enhancements [2]. However, other parameters, such as the instructions on how to communicate adequate information for improving performance, are key to a successful training program [3].

Cues are important within the training context for both sedentary and highly active individuals and may play a pivotal role in directing attention towards a specific way that affects motor performance or motor learning [4]. In fact, instructions facilitate the shifting of attention through an explicit thought during a specific task that is executed thanks to conscious or sub-conscious abilities [3]. With this in mind, an individual’s focus could be directed externally (i.e., external focus, EF) with metaphors or analogies (i.e., “explode by jumping high like a rocket”) [5] considering the effects of a specific movement on the environment, or internally (i.e., internal focus, IF) when the main focus is directed on specific body parts during movement (i.e., “flex your elbow at 90°”).

Fitness coaches and therapists would possibly benefit from a deeper knowledge about the differences in using external or internal focus strategies with their patients or athletes in order to improve performance.

There is evidence demonstrating the influence of attentional focus on various motor performance outcomes (i.e., postural control, muscle strength and speed) [6]. In particular, an external focus seems to be more effective for enhancing balance and postural control as compared to an internal focus [7]. Decreased postural sway when using an external cue was also noted when the level of balance difficulty was increased [8].

The benefits of using a specific attentional focus strategy have been demonstrated to be useful also during neuromuscular activities, indicating equivalent results between IF and EF modalities. On one hand, an external focus appears to be superior in a variety of isometric and dynamic tasks, showing higher strength production [9,10,11] and neuromuscular efficiency [11,12,13,14,15]; on the other, an internal focus condition induces a greater advantage in electromyographic activity during both upper- and lower-body muscular strength exercises [11,16]. These findings support the notion of promoting both attentional focus strategies for a better “mind–muscle” connection during resistance training exercises [17].

In recent years, these two attentional focus conditions (IF and EF) have gained even more interest among individuals with different health conditions [18,19,20,21,22,23,24,25,26].

In this regard, it has been demonstrated that an external instruction induced a greater improvement compared to an IF cue in speed and movement time in visually impaired individuals [18]. On the same line of evidence, positive results using an EF cue were also confirmed in idiopathic Parkinson’s disease patients across different performance outcomes such as postural sway, body balance and walking step length [19,20]. Stroke patients showed significant improvements in movement time performance in reaching, grasping or manipulating objects when following an external focus strategy [21,22].

Finally, similar results in using an EF cue compared to IF in enhancing performance outcomes were also noted in individuals with cardiac conditions, cancer, multiple sclerosis or cerebral palsy conditions [23,24,25,26].

Obesity is a complex health disease that alters the center of mass displacement [27], necessitating biomechanical adaptations to maintain body balance [28] and leading to a reduction in muscular strength, motor function and everyday-life activities [29]. Uncomfortable bodily sensations may account for decreased physical activity, but shifting attention towards a specific direction appears to be beneficial in increasing exercise practice [30], underlining “where” the attention should be centered while performing a given task [31].

Nevertheless, individuals with obesity display a lowered quality of movement performance [32], with a concomitant increase in physical inactivity [33], so the specific use of an attentional focus modality may play a role in increasing movement effectiveness [5]. To the best of our knowledge, no previous research analyzed the role of attentional focus in movement performance in subjects with obesity.

Therefore, the aim of this study was to evaluate the effects of different attentional focus strategies on functional movement performance, body balance, upper- and lower-body muscular strength and body composition in outpatients with obesity.

## 2. Materials and Methods

### 2.1. Participants

The present research involved Caucasian, obese outpatients who were referred to the IRCCS Istituto Auxologico Italiano, a specialized center for obesity weight management. The exclusion criteria were an age over 65 years, knee or hip pain (visual analogue scale score >7 arbitrary units), a history of hip, knee or foot replacement or osteoarthrosis, cardiac infarction, neurological impairments or any other clinical condition that affects the practice of physical activity. One hundred and thirty patients were screened. Twenty-eight of them (20%) did not reach the inclusion criteria, and eight patients dropped out (8%) during the intervention period. Thus, the final sample comprised 94 eligible individual who were randomized into IF and EF groups. No other adverse event was observed during the study.

### 2.2. Multidisciplinary Rehabilitation Program

The multidisciplinary rehabilitation program consisted of weekly visits for medical assessments, nutritional education, peer group psychological support and advice reinforcement regarding physical exercise.

A self-monitored diary including daily food consumption, physical exercise performed and emotional reactions was used as a tool for education and reinforcement.

A registered dietician provided to each subject a diet based on a 750 kcal/day deficit from the subject’s estimated caloric requirement (1200–2000 kcal/day). Specifically, each diet was composed of 17–22% protein, 23–25% fat and 55–58% carbohydrates.

### 2.3. Procedures

Inside the multidisciplinary rehabilitation program, each patient was randomly allocated into one of two intervention groups using a computer-based random number generator as follows: Internal Focus group [IF, n = 47] and External Focus group [EF, n = 47]. The researcher (LG) generated the random allocation sequence, and two other investigators (MC and GR) enrolled participants. In detail, each patient followed an individualized, home-based training program for 6 consecutive weeks (total training sessions: 18). During this period, each subject did not perform any other physical activity beyond the prescribed training program. Baseline and postintervention fitness parameters and anthropometric and body composition variables were recorded.

#### 2.3.1. Fitness Parameters

Functional performance was assessed using the Functional Movement Screen (FMS) developed by Cook and Burton [34,35].

It is composed of seven locomotor patterns, but to overtake movement difficulties presented by obese individuals, only the deep squat, the hurdle step, the shoulder mobility and the active straight leg raise patterns were used [36]. Finally, movement efficiency was scored using a 0-to-3-point scale focused on the quality of movement execution, providing a final composite score (the summation of each single score). A certified FMS instructor administrated the testing procedure. It has been demonstrated that the FMS has moderate–high inter–intra-rater reliability coefficients (Kappa coefficient = 0.64, 0.57, 0.76 and 0.79) [36].

The modified version of the Balance Error Scoring System (M-BESS) was employed to assess body balance [37]. Each patient was instructed to preserve a stable position for 20 s, with their eyes closed, during three stances on a firm surface while maintaining their hands on their hips (“bipodalic” with feet together, “tandem” with nondominant foot behind the dominant one or “monopodalic” on the nondominant foot). During each trial, the number of errors was counted in accordance with the referred protocol [38,39]. The M-BESS composite score was obtained by summing the total errors recorded. This testing procedure presents a high inter-rater reliability (ICC = 0.88) [37].

The Handgrip Strength Test (HST) is a valid and reliable (r = 0.81) measure of maximum isometric strength [40] and was used with the hydraulic dynamometer Jamar (J. A. Preston Corporation, Clifton, NJ, USA). The grip strength was detected three times for each hand alternately, starting with the dominant hand, with the patient in a sitting position with their elbows fixed at a 90-degree angle. The patient was asked to maximally squeeze the dynamometer for three consecutive seconds. The mean value was calculated for statistical analysis.

The Five-Repetition Sit-to-Stand (FRSTS) test was performed to evaluate lower-limb strength, showing a high intra-rater reliability coefficient (ICC = 0.81) [41,42]. The patient has to start in a sitting position with crossed arms over the chest. At the signal “ready, go”, provided by an expert investigator, the subject has to rise to a full standing position and return to the initial sitting position for five consecutive repetitions while the movement time execution is recorded. Three measurements using a manual stopwatch, observing a 60 s rest in between them, were performed.

#### 2.3.2. Body Composition and Anthropometric Parameters

A bioelectrical impedance assay (BIA 101-RJL Systems Akern srl, Firenze, Italy) was performed by trained nurses to assess the body composition after an overnight fast and with an empty bladder in supine position with the limbs slightly away from the body (30–45°), detecting the fat mass percentage (FM%).

Anthropometric assessment was conducted, measuring body height to the nearest 0.1 cm with a stadiometer (SECA^®^ 240, Hamburg, Germany) and body weight with a calibrated weight scale to the nearest 0.1 kg (SECA^®^ 877, Hamburg, Germany); body mass index (BMI) was calculated using the standard formula of weight (kilograms) divided by the square of height (meters squared), for which “obesity class I” is a value from 30 to 34.9 kg/m^2^, “obesity class II” is a value from 35 to 39.9 kg/m^2^, and “obesity class III” is a value greater than 40 kg/m^2^ [43].

### 2.4. Training Intervention

The home-based training protocol consisted of a 6-week exercise program designed to improve fitness levels and quality of movement (i.e., mobility and stability) as proposed in previous studies on individuals with disabilities or with other secondary health conditions such as obesity [32,44].

The intervention program was the same for both groups in terms of exercise selection, order, load, sets, repetitions and equipment, except for the verbal instructions, which shifted attention into an external or an internal environment. Specifically, a circuit-training modality was proposed (time of work per set: 30 s, time of rest between each exercise: 15 s, time of rest between sets: 2 min and total volume: 3 sets) for a duration of 45 min with a frequency of three days per week (Monday, Wednesday and Friday at 17:00 pm CET, GMT Rome). In order to equate the training intensity, the OMNI-res perceived exertion scale was used by setting a cut-off score of 6 points out of 10 [45].

In detail, the IF group followed an internal cue directing attention to the specifics of movement (i.e., joint motion, with a single body part involved), while the EF group focused attention on the global process of an action by using analogies or metaphors. A summarized description of instructions and cues proposed is shown in Table 1.

The training adherence was recorded weekly by a certified sport scientist who verified the correct exercise form together with the daily diary compilation about home training (i.e., training duration in minutes and the rating of perceived exertion using the RPE, CR-10 scale).

### 2.5. Statement of Ethics

The study was ethically conducted in accordance with the World Medical Association Declaration of Helsinki. Patients provided their written informed consent, and the study protocol was approved by the Ethics Committee of the IRCCS Istituto Auxologico Italiano (approval number 2020_09_29_01). This research was in compliance with the TIDieR checklist [46].

### 2.6. Statistical Analysis

Data are expressed as mean ± standard deviation, and the normality of the distribution was checked using the Kolmogorov–Smirnov and Shapiro–Wilk tests. An unpaired *t*-test was performed to examine potential baseline differences in both groups. Sample size calculation was established via power analysis (α = 0.05, β = 0.75, effect size = 0.78) on the basis of a previous study examining the effects of movement quality training on obese individuals [32]. A two-way repeated measure analysis of variance (ANOVA RM) was used to investigate the interactions (time x intervention) and main effects of time (pre/post treatment) and groups (IF and EF group) regarding fitness, body composition and anthropometric parameters. In the case of significance, Bonferroni post-hoc analysis was used. Partial eta squared (Part η2) effect size was used to estimate the magnitude of the difference within each group, and thresholds for small, moderate and large effects were defined as 0.01, 0.06 and 0.14, respectively [47]. To detect between-group gender differences (males IF group vs. males EF group and females IF group vs. females EF groups), the Mann–Whitney U test was performed. Finally, the minimal clinically important difference (MCID) was provided by multiplying the standard deviation (SD) of the baseline scores by 0.2 (i.e., MCID = 0.2 × SD) [48] to check for potential clinically meaningful change. All statistical analyses were performed using the Statistical Package for Social Sciences, IBM™ SPSS™ Statistics (version 21.0, IBM Corp., Somers, Chicago, IL, USA).

## 3. Results

At the baseline, the mean age, sex, body mass, FM% and functional parameters did not show any statistical significance (*p* > 0.05) for the two groups (Table 2).

**Figure 1:**
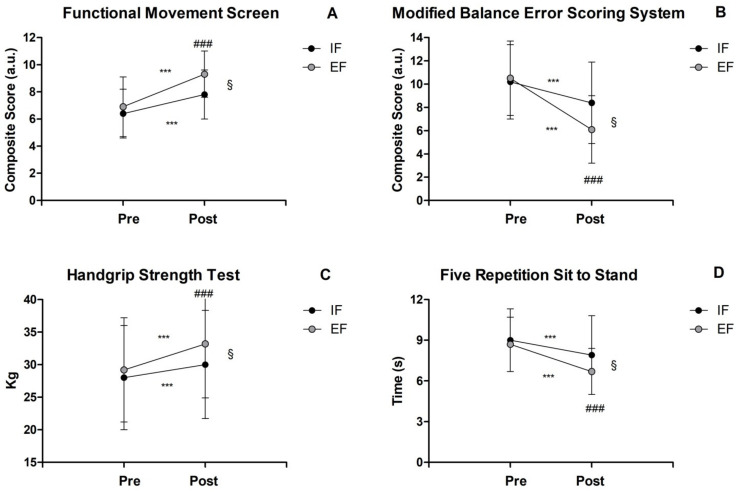
Fitness parameters: (**A**) FMS Composite Score, (**B**) M-BESS Composite Score, (**C**) HST, (**D**) FRSTS. ^§^ Significant interaction (*p* < 0.001). *** Significant main effect of time before and after testing (*p* < 0.001). ^###^ Significant main effect of group (EF vs. IF) (*p* < 0.001).

Regarding the FMS test, the composite score demonstrated an improvement by 35% (6.9 ± 1.8 a.u. pretraining vs. 9.3 ± 1.8 a.u. posttraining) in EF and 21% in IF (6.5 ± 1.9 a.u. pretraining vs. 7.8 ± 2.2 a.u. posttraining), with a significant interaction (F_1,26_ = 15.6, *p* ≤ 0.001, part η2 = 0.68) (Figure 1A). Additionally, significant main effects of time (pre-to-post) (F_1,26_ = 15.6, *p* ≤ 0.001, part η2 = 0.14) and of group (F_1,26_ = 1658.6, *p* ≤ 0.001, part η2 = 0.94) were found. Moreover, it should be noted that the EF group effect size (d = 1.3) was superior to the MCID value of 0.4.

Concerning the M-BESS composite score, there was an improvment by 42% in the EF group (10.5 ± 3.3 a.u. pretraining vs. 6.1 ± 2.9 a.u. posttraining) compared to the IF group (18%, from 10.2 ± 3.3 a.u. to 8.4 ± 3.5 a.u.), with a significant interaction (F_1,26_ = 123.7, *p* ≤ 0.001, part η2 = 0.57) (Figure 1B) and concomitant main effects of time (F_1,26_ = 21.5, *p* < 0.001, part η2 = 0.19) and group (F_1,26_ = 829.5, *p* ≤ 0.001, part η2 = 0.90). Notably, it should be remarked that only the EF group effect size (d = 1.4) was clinically meaningful compared to the related MCID score (0.7).

Regarding the grip strength, the HST revealed an increase by 13% in the EF group (from 29.3 ± 9.8 kg to 33.2 ± 9.7 kg) compared to 7% in the IF group (from 28.0 ± 8.0 kg to 30.0 ± 8.4 kg), with a significant interaction (F_1,26_ = 227.8, *p* ≤ 0.001, part η2 = 1.00) as well as main effects of time (F_1,26_ = 24.0, *p* ≤ 0.001, part η2 = 0.99) (Figure 1C) and group (F_1,26_ = 1062.1, *p* ≤ 0.001, part η2 = 1.00). Moreover, both groups showed a lower effect size (IF d = 0.2; EF d = 0.4) than the expected MCID value (IF = 1.6; EF = 2.0).

Concerning the FRSTS test, the EF group exhibited an improvement by 23% (8.8 ± 2.1 s pretraining vs. 6.7 ± 1.8 s posttraining) compared to 12% in the IF group (9.0 ± 3.0 s pretraining vs. 8.2 ± 2.3 s posttraining), with a significant interaction (F_1,26_ = 155.7, *p* ≤ 0.001, part η2 = 1.00) as well as main effects of time (F_1,26_ = 14.5, *p* ≤ 0.001, part η2 = 0.96) and group (F_1,26_ = 1205.2, *p* ≤ 0.001, part η2 = 1.00) (Figure 1D). It should be also highlighted that the EF group effect size (d = 1.0) was clinically meaningful compared to the referred MCID value of 0.4.

**Figure 2:**
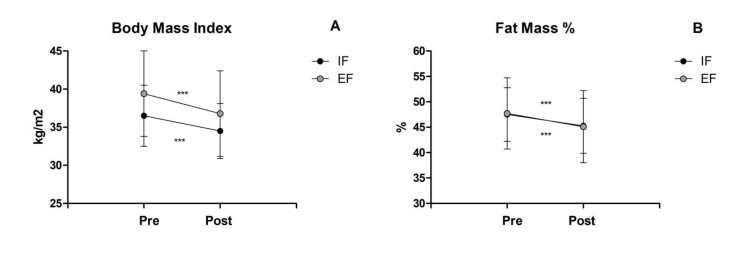
Anthropometric and body compositional parameters: (**A**) Body Mass Index, (**B**) Fat Mass percentage. *** Significant main effect of time before and after testing (*p* < 0.001).

Finally, both the fat mass percentage and body mass index did not reveal any significant interaction (Figure 2A,B) except for a main effect of time (FM%, F_1,26_ = 5233.0, *p* ≤ 0.001, part η2 = 1.00; BMI, F_1,26_ = 194.1, *p* ≤ 0.001, part η2 = 1.00). In detail, the EF and IF groups showed a similar decrease by 5% in FM% (EF from 47.7 ± 4.2 % to 45.1 ± 7.1 %; IF from 47.5 ± 5.3 % to 45.4 ± 5.4 %), as well as a reduction in BMI (EF 6%, from 37.7 ± 3.9 kg/m^2^ to 35.4 ± 4.2 kg/m^2^; IF 5%, from 36.5 ± 4.0 kg/m^2^ to 34.6 ± 3.7 kg/m^2^).

Lastly, between-group gender comparisons showed significant differences in all fitness parameters (*p* < 0.05) in favor of males of the EF group compared to their IF counterparts, as well as for females in the EF group with respect to their IF peers.

## 4. Discussion

This study investigates the effectiveness of a 6-week home-based intervention designed with an internal or an external focus that provides instruction to individuals with obesity. To the best of our knowledge, the present study is the first that analyzed different attentional focuses in improving motor performance and body composition among outpatients with obesity.

In particular, the main finding we observed was that the EF group performed significantly better than the IF group in the FMS, M-BESS, HST and FRSTS tests after the intervention period in both sexes. Interestingly, as opposed to fitness parameters, the EF and IF groups responded similarly to training in anthropometric and body composition outcomes.

As for the Functional Movement Screen and the Modified Balance Error Scoring System composite score, it is possible to appreciate that an external focus (EF) allowed significant improvements in functional performance and body balance, with subjects whose intervention used such a focus exhibiting a higher movement efficiency and stability compared to the IF group.

In line with this, previous evidence highlighted that an external focus is more effective in improving balance and postural control, as shown by decreased postural sway, [7,23]. In addition, external cues appear to be superior also at higher levels of difficulty of balance exercises, with patients showing reduced body oscillations when stability is requested to preserve the base of support [8]. Because excessive body weight may alter the center of mass displacement, with a concomitant reduction in lateral stability imposing postural adaptations, individuals with obesity may benefit from increased functional performance and body balance [28,49].

As for upper- and lower-body muscular strength, both the HST and the FRSTS results observed in the EF group highlighted significant improvements after the 6-week training intervention. There is evidence that a short-term period (i.e., six weeks) [50] of an external cue intervention is sufficient to provide positive effects on maximal handgrip strength, isometric mid-high pull, squat and deadlift tests as compared to an intervention with an internal focus [51,52,53]. With this in mind, a resistance training program could be beneficial when dealing with obesity because when muscular strength is normalized to body mass, obese patients are weaker than their normal-weight counterparts. This relative weakness may be due to lowered neural adaptation or changes in muscle morphology [54] inducing consequences in health status, especially in individuals with sarcopenic obesity [55].

Finally, observing the anthropometric and body composition parameters, fat mass percentage and body mass index demonstrated significant pre-to-postintervention changes, with no differences between groups on baseline (*p* > 0.05). To the best of our knowledge there have been no randomized controlled trials that aimed to confirm the superiority of an external focus strategy regarding body composition or nutritional parameters.

In a recent study, Bilman et al. [56] observed that external cues related to food (i.e., packaging, portion sizes and labeling) may have an effect on subjective feelings of hunger and satiety, facilitating or hampering the internal signal management related to food intake regulation. From this perspective, for a better awareness of food consumption and regulation, it is important to structure the entire process of following external cues into five phases (i.e., meal initiation, meal planning, consumption phase, end of eating episode and time until next meal) [56].

In summary, from a general perspective, a plausible explanation for the benefits of adopting an external rather than an internal focus during motor performances is the constrained action hypothesis [57]. This theoretical viewpoint implies that, when an individual directs their attention externally, the motor control system self-organizes more effectively, facilitating automatic, non-conscious motor control, with the result of a more fluent and regular movement execution. On the contrary, in the case of an internal focus strategy, the motor control system becomes more constrained, with the direct consequence of depressing motor performance due to more controlled and conscious movement [57]. Moreover, when using an EF, a more economic strategy is possible because it seems to facilitate altered neural activation de facto, resulting in more intracortical inhibition that reduces the upper motoneurons’ activation, accompanied by a reduced slower motor pathway [14,58].

One of the strengths of this study is that the training program structured on external focus feedback seems to be superior in improving functional performance, balance and muscular strength in a short time period. This was the first study to use an outpatient population with obesity in an attentional focus study of motor performance. Our results need to be replicated before drawing conclusions about the proper way to instruct this type of individual. However, as demonstrated in subjects with different health conditions, our results support the use of an external focus strategy when managing subjects with obesity in improving fitness levels and efficiency of movement.

Finally, from a practical point of view, we could speculate that fitness coaches or therapists could benefit from designing their training programs using an external focus, not only with individuals with obesity in a gym-based setting, but also online in the context of telerehabilitation [59].

The current study has several limitations. First, the lack of a control group following a neutral focus instruction makes it difficult to generalize our results. Second, it is not possible to control a participant’s attentional focus; frequent reminders and standardized procedures were used to maximize adherence, but there is always the possibility that an individual may use a focus that is different from what was prescribed. Third, the exercise training intervention was customized inside a multidisciplinary approach in which nutritional and psychological support were also present, so it is not possible to exclude the role of these aspects. Fourth, there was a lack of computational intelligence analysis, frequently used in biomedicine and sport contexts, to monitor individuals’ fitness levels [60]. Fifth, gender differences may have accounted for balance or strength results in response to the training interventions.

Future research should investigate how attentional focus manipulation (i.e., external, internal or neutral) influences the perception of exertion and motor performance during a specific form of endurance training, an exercise modality particularly tailored to counteract obesity.

## 5. Conclusions

The current study highlighted preliminary data on short-term effects in using a specific attentional focus strategy in obese outpatients who performed a 6-week home-based training program. In particular, this study indicates that an external focus can significantly improve functional movement performance, body balance, and upper- and lower-body muscular strength in obese outpatients.

The present findings suggest that emphasis should be placed on external instruction in order for such instruction to have positive influences on motor performance.

Our results provide initial evidence for fitness coaches or therapists of the usefulness of integrating different attentional focus strategies when designing rehabilitation programs.

## Figures and Tables

**Table 1:** Instructions performed by IF and EF groups.

Name of Exercise	IF Cue	EF Cue
Yoga Triangle Pose	Keep your feet wider than shoulder-width apart, extend one elbow overhead and bend to the side the spine at the hip joint and then return to the starting position.	Open your legs laterally, push one arm to the sky and then bend to the side the spine like a half-moon and then return to the starting position.
Wall Lat stretching	Facing the wall and staying a foot away, flex forward your spine at the hip joint, putting your hands on the wall.	Facing a wall and staying a foot away, lean forward your spine, gently putting your hands on the wall while imagining moving the wall away.
In place standing marching	Stand with your feet slightly apart with arms at the side; then, start to step in place, lifting your knees and moving your arms, both flexed at 90° at the elbow.	Stand with your feet slightly apart with arms at the side; then, start to step in place as if you are a puppet; please swing your arms like a pendulum.
Goblet Squat with dumbbell	Stand with your feet shoulder-width apart, then go down in a squatting position by bending your hips and knees at 90° flexed.	Stand with your feet shoulder-width apart, then go down with your back by imagining sitting on an invisible stool behind you.
Dumbbells curl to press	Flex simultaneously both elbows to the shoulders, rotate your palms outwards and then extend both elbows overhead.	Curl both arms simultaneously like a hook and then push them gently to the sky by imagining putting a suitcase on a shelf.
In place forward lunges	Start with feet hip-width apart, then take a big step forward with one leg by flexing your knees at 90° until the thigh is parallel to the floor and the shin is vertical.	Start with feet hip-width apart, then take a big step forward with one leg and bend both knees as if you are picking up two suitcases from the ground.
Wall push-up with torso rotation	Place both hands on the wall at shoulder height, slightly wider than your shoulders; then, take a step backward with both feet. From here flex both elbows approaching the sternum toward the wall. Finally, push yourself away from the wall, reaching the starting position, and begin to externally rotate the torso.	Place both hands on the wall at shoulder height, then take a step backward with both feet. From here, bend your arms, lowering the upper-body toward the wall. Finally, push yourself away from the wall as if you want to push someone away and begin to rotate your body outward as if you are screwing in a light bulb.
One-Arm overhead dumbbell extension from staggered stance	Hold a dumbbell in one hand behind your head, with your elbow flexed and pointed toward the ceiling. Then, extend your elbow overhead until the arm is straight with the dumbbell directly above you.	Hold a dumbbell in one hand behind your head, with your arm bent. Then, gently extend your arm to the sky by imagining hammering a nail.

**Table 2:** Baseline characteristics for IF and EF groups.

Characteristics	IF, n = 47	EF, n = 47
Participants’ Age (years)	54.3 ± 10.1	53.9 ± 10.4
Sex (Male/Female)	8/39	9/38
Body Mass Index (kg/m^2^)	36.5 ± 4.0	37.7 ± 3.9
Fat Mass (%)	47.5 ± 5.3	47.7 ± 4.2
Functional Movement Screen (a.u.)	6.5 ± 1.9	6.9 ± 1.8
Modified Balance Error Scoring System (a.u.)	10.2 ± 3.3	10.5 ± 3.3
Handgrip Strength Test (kg)	28.0 ± 8.0	29.3 ± 9.8
Five-Repetition Sit-to-Stand test (s)	9.0 ± 3.0	8.8 ± 2.1

A paired *t*-test was performed to examine potential baseline differences in both groups with no significant differences between each item of the two groups (*p* > 0.05). a.u. = arbitrary units, kg = kilograms, s = seconds.

## Data Availability

The datasets used during the current study are available from the corresponding author upon reasonable request.
